# Predictive Value of EGFR Expression for the Efficacy of Near-Infrared Photoimmunotherapy in Head and Neck Cancer

**DOI:** 10.31662/jmaj.2025-0174

**Published:** 2025-11-21

**Authors:** Shinsuke Suzuki, Yukie Taguchi, Haruka Kaya, Takuro Kitabayashi, Riko Asano, Yui Miyabe, Nobuko Sato, Yohei Kawasaki, Hiroshi Nanjo, Takechiyo Yamada

**Affiliations:** 1 Department of Otorhinolaryngology & Head and Neck Surgery, Akita University Graduate School of Medicine, Akita, Japan; 2 Division of Surgical Pathology, Akita University Hospital, Akita, Japan

**Keywords:** near-infrared photoimmunotherapy, head and neck cancer, epidermal growth factor receptor

## Abstract

**Introduction:**

Near-infrared photoimmunotherapy (NIR-PIT) has emerged as a promising treatment for unresectable locally advanced or recurrent head and neck cancer. This study aimed to identify potential predictors of NIR-PIT efficacy before treatment by focusing on blood biomarkers in addition to pathological findings, including epidermal growth factor receptor (EGFR) expression in tumors.

**Methods:**

A retrospective analysis of the medical records of 10 patients with head and neck cancer, who exhibited confirmed EGFR expression and underwent NIR-PIT treatment at Akita University Hospital from December 2021 to April 2024, was conducted (13 cycles of NIR-PIT). EGFR expression, cluster of differentiation (CD)4/CD8 ratio, regulatory T cell (Treg) frequency, serum albumin, neutrophil-to-lymphocyte ratio (NLR), and neutrophil-to-eosinophil ratio (NER) were calculated from the tumor tissue and blood collected immediately before treatment. Correlations of these factors with tumor response to NIR-PIT were determined.

**Results:**

The objective response rate (ORR) was 61.5% and the disease control rate (DCR) was 100%. A statistically significant association was observed between the EGFR index and tumor response. No statistically significant correlation was found between other biomarkers (CD4/CD8 ratio, Treg frequency, serum albumin, NLR, NER) and tumor response.

**Conclusions:**

These findings underscore the important role of EGFR expression in predicting the efficacy of NIR-PIT in the management of head and neck cancer, and highlight the significance of incorporating EGFR assessment in patient selection and optimized treatment strategies. Further studies are needed to elucidate the role that these other potential predictors, including tumor immune response markers, play in NIR-PIT outcomes.

## Introduction

Near-infrared photoimmunotherapy (NIR-PIT) is an innovative cancer treatment that combines the specificity of antibody-targeting with the cytotoxic effects of NIR light. This treatment consists of a tumor-targeting monoclonal antibody conjugated to a phthalocyanine dye, which selectively binds to cancer cells. Upon exposure to NIR light, the dye is activated, resulting in localized cell death and the induction of immunogenic cell death (ICD), which can stimulate a broader immune response against the tumor ^(1)^.

NIR-PIT has been administered in Japan for treating unresectable locally advanced or recurrent head and neck cancers since its introduction in 2021. For head and neck cancer treatment, this therapy consists of a conjugate of the light-absorbing dye IRDye700DX (IR700) and an antibody, specifically targeting the epidermal growth factor receptor (EGFR), which is overexpressed in a majority of head and neck squamous cell carcinomas (HNSCC) ^(2)^.

Clinical studies have yielded promising results, with high response rates and manageable adverse events ^(3), (4)^. The tumor-specific nature of NIR-PIT and its potential to induce immune responses make it a promising option for locoregional recurrent or metastatic disease ^(4)^.

The accurate prediction of treatment outcome is paramount in clinical oncology. The identification of patients likely to benefit from a specific therapy facilitates optimized treatment planning, minimizes unnecessary complications, and improves resource allocation ^(5)^.

Many factors have been evaluated as predictors of treatment efficacy in various cancers, including patient Eastern Cooperative Oncology Group (ECOG) performance status (PS) ^(6)^, peripheral blood parameters ^(7), (8)^, and tumor characteristics, such as the expression of molecular markers ^(9)^.

NIR-PIT is likely influenced by various factors, including its mechanism of action, target molecule expression, and intratumoral immune responses ^(10), (11)^. Previous studies indicate that the neutrophil-to-lymphocyte ratio (NLR) before NIR-PIT treatment can predict treatment outcome ^(12)^; however, factors that predict NIR-PIT efficacy before treatment initiation remain unclear.

Because of the important role of EGFR in the mechanism of NIR-PIT in head and neck cancer, we hypothesized that EGFR expression within the tumor significantly influences treatment outcomes. However, the clinical significance of EGFR expression in predicting NIR-PIT efficacy in this patient population has not been evaluated. Therefore, to address this knowledge gap, we conducted a study to identify potential predictors of NIR-PIT efficacy before treatment, focusing on both tumor tissue and peripheral blood data collected from patients with head and neck cancer.

## Materials and Methods

### Patients and data collection

We reviewed the medical records of consecutive head and neck cancer patients with SCC or non-SCC with confirmed EGFR expression, who were treated with NIR-PIT at the Department of Otorhinolaryngology-Head and Neck Surgery, Akita University Hospital (Akita, Japan) from December 2021 to April 2024, in which treatment for tumor size could be measured by imaging before treatment and pathological evaluation of the tissue was available.

This study was approved by the Institutional Review Board of Akita University Hospital (No. 3154) and conducted according to the principles of the Declaration of Helsinki. Because of the retrospective nature of the study, the requirement for patient informed consent was waived; however, patients were allowed to decline the use of their clinical records for research (opt-out consent provision). The study began on September 1, 2024, and participant data were collected from the beginning of the study to December 1, 2024, which was the cutoff date. All patient data were coded to ensure the privacy of the subjects and data confidentiality.

Tumor response was assessed according to the Response Evaluation Criteria in Solid Tumors, version 1.1. The objective response rate (ORR) was defined as the proportion of patients who exhibited complete response (CR) or partial response (PR) as the best response. The disease control rate (DCR) was defined as the proportion of patients who exhibited a CR, PR, or stationary disease (SD) as the best response. The clinical response to treatment was evaluated using computed tomography every 4-12 weeks. Progression-free survival (PFS) was defined as the time from each cycle of NIR-PIT until disease progression, death from any cause, or the cutoff date if no progression occurred. Overall survival (OS) was defined as the time from the initiation of the first NIR-PIT until death from any cause or the cutoff date.

### Measurement of the peripheral blood biomarkers

The following parameters were simultaneously measured from complete blood count measurements obtained 1-3 days before each NIR-PIT treatment: serum albumin (Alb), NLR, and neutrophil-to-eosinophil ratio (NER). NLR was defined as the ratio of the neutrophil count divided by the lymphocyte count. NER was calculated by the neutrophil count divided by the eosinophil count.

### Immunohistochemistry and classification of pathological findings

**Figure 1.**
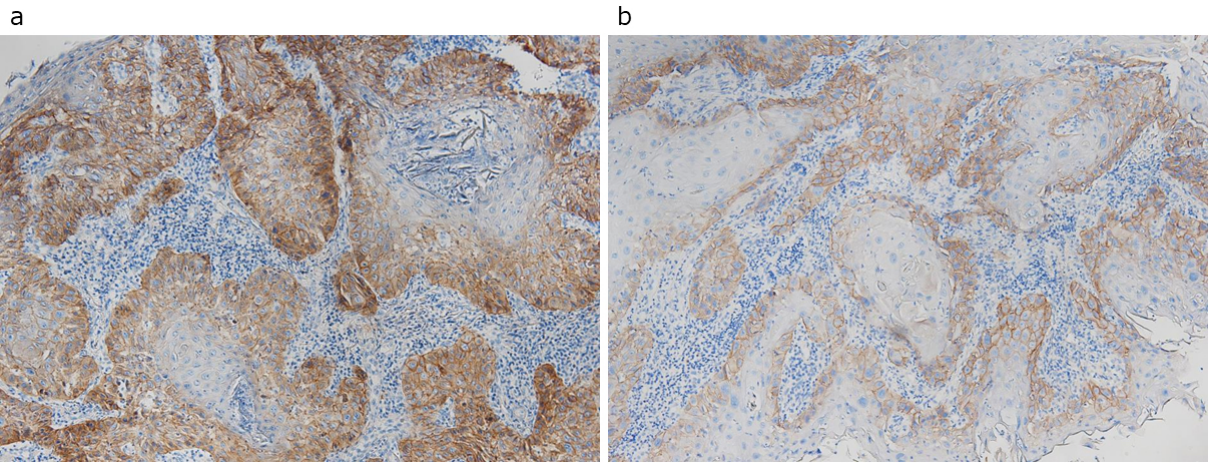
EGFR expression in tumor. Immunohistochemical analysis of (a) Index = 4 (positive area, 2; intensity, 2) and (b) Index = 1 (positive area, 1; intensity, 1) specimens.

Tumor tissues collected immediately before NIR-PIT treatment were fixed with 10% neutral buffered formalin, and consecutive sections were cut every 5 mm and 4-μm thick. The sections were stained with hematoxylin and eosin. Polyclonal EGFR, cluster of differentiation (CD)4, CD8, and forkhead box P3 (Foxp3) antibodies were used for immunohistochemical staining. The identification of regulatory T cell (Treg) was performed by the expression of CD4 and Foxp3. The tumor microenvironment was examined, and the CD4/CD8 ratio and Treg frequency of the infiltrating lymphocytes was calculated. EGFR expression was scored based on a previous report ^(13)^. Briefly, EGFR staining areas received a score of 0 if <10% of cells in the tumor nest were stained, a score of 1 for ≥10% and <50% were stained, and a score of 2 if ≥50% were stained. The EGFR staining intensity was also scored from 0 to 2, and an EGFR index was calculated (range: 0-4) as EGFR-positive area (0-2) × EGFR intensity (0-2) (Figure 1).

### Statistical analysis

A two-tailed Mann-Whitney U test was used to assess the statistical significance of differences in the variables of each parameter and the response to NIR-PIT. The Spearman’s rank correlation coefficient was used to assess the significance of the correlation between each parameter and PFS. A p <0.05 was considered statistically significant. The statistical analyses were performed using EZR, version 4.0.0 (Saitama Medical Center, Jichi Medical University, Saitama, Japan).

## Results

### Patient characteristics

In Japan, NIR-PIT treatment may be repeated for up to four cycles. During the study period, a total of 13 NIR-PIT cycles were performed on 10 patients. Six received one cycle, and four received two cycles with NIR-PIT. Patient demographics and respective backgrounds are listed in Table 1. The cohort included nine male patients and one female patient. The median age at the initial NIR-PIT treatment was 73 years (range: 54-83 years). All patients exhibited an ECOG PS of 0 and had a history of chemoradiotherapy. The median tumor volume was 3,840 cm^3^ (range: 600-26,400 cm^3^).

**Table 1.** Clinical Characteristics and Clinical Outcome of 10 Patients Treated With NIR-PIT.

Case	Cycle	Gender	Age	ECOG PS	Primary site	Previous treatment	Therapeutic target site	Histology (differentiation in the case of SCC)	EGFR index	CD4/CD8	Treg frequency (%)	NLR	NER	Albumin (g/dL)	BOR (best overall response	Treatments following NIR-PIT	OS (days)	PFS	Outcome
1	1	Male	73	0	Tongue	Surgical operation, Chemoradiation	local recurrence	SCC (well)	4	3	50	2.9	13.7	4.2	PR	NIR-PIT	1096	31	Alive
	2							SCC (well)	1	3	10	4.4	8.0	3.8	SD	Surgical operation		44	
2	1	Male	74	0	Hypopharynx	Surgical operation, Chemoradiation	lymph node metastasis	SCC (moderate)	1	4	5	39.3	1180.0	3.4	SD	BSC	210	55	Death
3	1	Male	73	0	Oropharynx	Chemoradiation	local recurrence	SCC (moderate)	4	2	10	4.6	37.0	3.9	PR	Surgical operation	786	117	Alive
4	1	Male	54	0	Ethmoid sinus	Chemoradiation	local recurrence	SCC (moderate)	0	9	0	1.9	190.0	2.7	SD	NIR-PIT	709	40	Alive
	2							SCC (moderate)	2	2	20	2.2	24.0	4.5	SD	Proton Beam Therapy		36	
5	1	Male	83	0	Oropharynx	Chemoradiation	local recurrence	SCC (well)	4	4	10	4.4	48.0	3.8	CR		695	695	Alive
6	1	Male	78	0	Lower gingiva	Surgical operation, Chemoradiation	local recurrence	sarcomatoid carcinoma	4	3	30	4.5	27.0	2.5	CR		667	667	Alive
7	1	Female	83	0	Parotid gland	Chemoradiation	local recurrence	SCC (well)	2	1	10	2.3	180.0	3.9	CR		543	543	Alive
8	1	Male	74	0	Oropharynx	Surgical operation, Chemoradiation	local recurrence	SCC (well)	4	2	10	4.0	14.7	3.7	PR	BSC	127	89	Death
9	1	Male	73	0	Oropharynx	Chemoradiation	local recurrence	SCC (moderate)	4	3	10	4.0	36.0	3.8	CR		359	359	Alive
10	1	Male	58	0	Hypopharynx	Surgical operation, Chemoradiation	skin metastasis	SCC (moderate)	2	3	20	1.5	160.0	4.5	SD	NIR-PIT	326	36	Alive
	2							SCC (moderate)	4	3	20	4.2	28.4	4.1	PR	NIR-PIT		26	

CD: cluster of differentiation; ECOG PS: Eastern Cooperative Oncology Group performance status; EGFR: epidermal growth factor receptor; NER: neutrophil-to-eosinophil ratio; NIR-PIT: near-infrared photoimmunotherapy; NLR: neutrophil-to-lymphocyte ratio; OS, overall survival; PFS: progression-free survival; SCC: squamous cell carcinoma; Treg: regulatory T cell.

It should be noted that the median (range) age and tumor volume were 73.5 years (58-83 years) and 5,148 cm^3^ (600-21,000 cm^3^) in the response group, and 58.0 (54-74) and 2925 cm^3^ (1,560-26,400 cm^3^) in the non-response group, respectively. No statistically significant differences were observed for either age (p = 0.073) or tumor volume (p = 0.833).

Histological diagnoses taken immediately before each treatment cycle revealed SCC in nine cases and sarcomatoid carcinoma in one case. EGFR expression was confirmed in all cases, with a median EGFR index (range) of 4 (0-4). For reference, in Case 4, although the EGFR intensity was 1, the EGFR-positive area was determined to be 0 because it was less than 10%. Consequently, the EGFR index was calculated as 0. Concurrently, the median (range) for the parameters calculated from blood samples obtained immediately before each treatment cycle were as follows: 3 (1-9) for CD4/CD8, 10% (0%-50%) for Treg frequency, 4 (1.9-39.3) for NLR, 36 (8-1,180) for NER, and 3.8 g/dL (2.5-4.5 g/dL) for albumin. The best overall response (BOR) for each treatment cycle was CR 4, PR 4, and SD 5, with an ORR of 61.5% and a DCR of 100%. Four patients (cases 5, 6, 7, and 9) had a CR following a single NIR-PIT treatment. Two patients (cases 1 and 3) exhibited tumor shrinkage following the initial round of NIR-PIT, rendering the lesions resectable by surgical intervention. Case number 4 did not achieve sufficient tumor shrinkage after two NIR-PIT treatments, and was subsequently treated with proton beam therapy. Case 10 showed PR following the second NIR-PIT treatment during the study period, subsequently undergoing two additional NIR-PIT treatments. All eight cases survived until the cutoff date. Two cases (2 and 8) were transitioned to best supportive care after the initial NIR-PIT because of a deterioration in their general condition, which was attributed to disease progression. These two patients ultimately succumbed within the observation period. As a result, the median OS from the initial NIR-PIT for each patient was 605 days (127-1,096), whereas the median (range) PFS for each NIR-PIT treatment was 55 days (31-695).

### Association of pathological findings and peripheral blood biomarkers with tumor response

**Figure 2.**
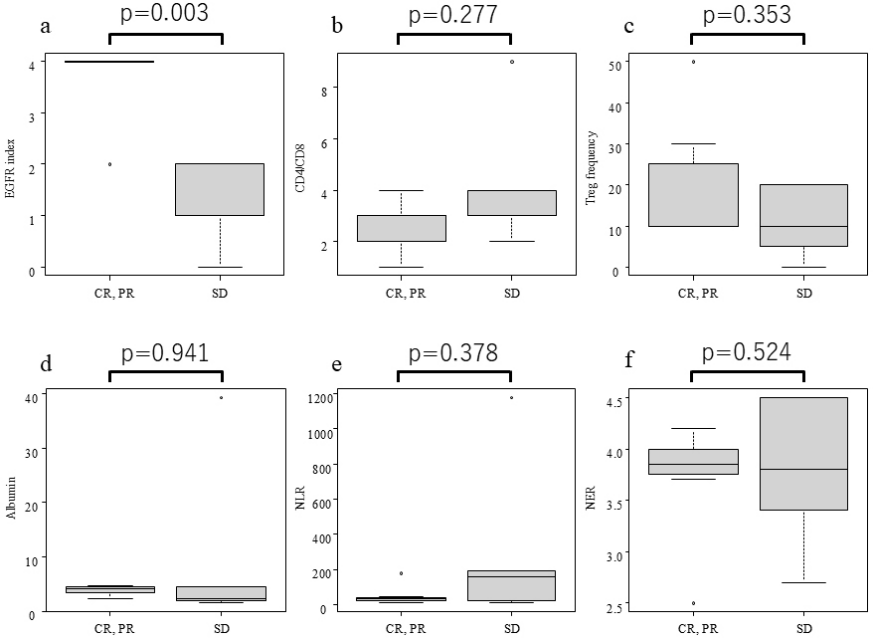
Box and whisker plots (median, first, and third quartiles, minimum and maximum) are used to represent pathological and blood parameters:(a) EGFR index, (b) CD4/CD8, (c) Treg frequency, (d) serum albumin, (e) NLR, (f) NER on responders (CR, PR) and non-responders (SD) of NIR-PIT treatment. p-Values are from Mann-Whitney U tests comparing means or medians between responders (CR and PR) and non-responders (SD). Significant p-values (p < 0.05) are marked with an asterisk (*). CD: cluster of differentiation; CR: complete response; EGFR: epidermal growth factor receptor; NER: neutrophil-to-eosinophil ratio; NIR-PIT: near-infrared photoimmunotherapy; NLR: neutrophil-to-lymphocyte ratio; PR: partial response; SD: stable response; Treg: regulatory T cell.

The relationship between BOR for each treatment cycle and histopathology and peripheral blood biomarkers collected immediately before each treatment cycle was examined. A statistically significant disparity between the response group (CR, PR) and the non-response group (SD) was observed in the median (range) of the EGFR index obtained from pathology [4 (2-4) vs. 1 (0-1)], yielding a p-value of 0.003. Conversely, the median (range) of the CD4/CD8 ratio and Treg frequency in the response and non-response groups, also obtained from pathology, were 3 (1-4) and 3 (2-9), and 10% (10%-50%) and 10% (0%-20 %), respectively, with no statistically significant differences. For the blood biomarkers examined in this study, the median values (range) of the response and non-response groups were: Alb: 4.1 g/dL (2.3-4.6 g/dL) and 2.2 g/dL (1.5-39.3 g/dL), respectively. The median values for the NLR were 32.2 (13.7-180) and 160 (8-1,180), whereas the median values for the NLR were 3.9 (2.5-4.2) and 3.8 (2.7-4.5), respectively. No statistically significant differences were observed in any of the cases (Figure 2).

### Association between the pathological findings, peripheral blood biomarkers, and PFS

**Figure 3.**
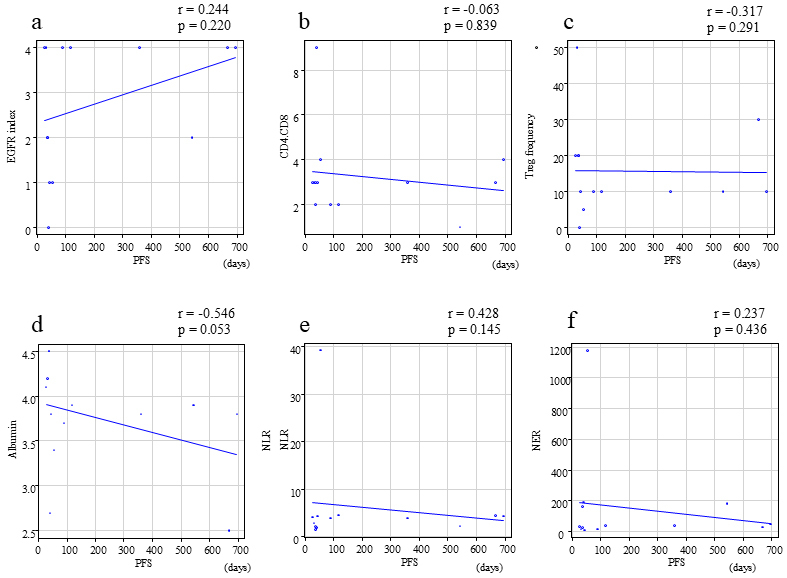
Correlation between (a) EGFR index, (b) CD4/CD8, (c) Treg frequency, (d) serum albumin, (e) NLR, (f) NER, and PFS. CD: cluster of differentiation; EGFR: epidermal growth factor receptor; NER: neutrophil-to-eosinophil ratio; NLR: neutrophil-to-lymphocyte ratio; PFS: progression-free survival; Treg: regulatory T cell.

The present study was primarily focused on determining the correlation between the response of tumors to NIR-PIT treatment and the factors derived from pathology and blood samples obtained immediately before each treatment cycle. Consequently, the focus was narrowed to solely assess the prognostic significance of PFS within each treatment cycle. The correlation between pathological factors and blood biomarkers immediately before each treatment cycle and PFS was evaluated, and the correlation coefficients (r) and p-values for each factor with PFS were as follows: EGFR index (r = 0.244, p = 0.22), CD4/CD8 (r = −0.063, p = 0.839), Treg frequency (r = −0.317, p = 0.291), serum albumin (r = −0.546, p = 0.053), NLR (r = 0.428, p = 0.145), and NER (r = 0.237, p = 0.436). No statistically significant correlation was observed between PFS and any of these factors (Figure 3).

## Discussion

The therapeutic principle of NIR-PIT is to destroy tumor cells by irradiating them with NIR light to which antigen-specific antibodies conjugated with light-sensitive substances have been attached ^(14)^. NIR-PIT results in the release of intracellular tumor antigens during the destruction of the target cells. Consequently, NIR-PIT induces ICD, thereby activating the host immune response to the tumor ^(15)^. A study of clinical specimens demonstrated that the immunophenotyping of whole blood revealed the systemic induction of innate and adaptive immunity (such as natural killer cells, cytokines, lymphocytes, monocytes, CD4, and CD8 T cells) by NIR-PIT ^(16)^. This suggests that the therapeutic efficacy of NIR-PIT is not solely attributed to direct physical cell destruction, but also encompasses the activation of immune cells.

The process of inducing immune cell activation enhances a series of antitumor immune capabilities, in which the host immune cells recognize and attack cancer cells. This antitumor immune capacity is required for the immune checkpoint inhibitor (ICI) to manifest its antitumor effect. NIR-PIT is believed to engender a conducive tumor microenvironment for ICI treatment ^(17), (18)^. Furthermore, antigen-specific antibodies used for NIR-PIT have the potential to enhance antibody-dependent cellular cytotoxicity, the mechanism through which the immune system eradicates cancer cells, thereby amplifying the therapeutic efficacy of ICI ^(19)^. The combination of these mechanisms may generate a potent synergistic effect, in which the PIT and ICI enhance the immune response to the tumor. Studies in murine models have demonstrated that the concurrent administration of NIR-PIT and ICIs result in the regression of distant, non-NIR-PIT-treated tumors, fostering optimism for the efficacy of these combination therapies ^(20)^.

The findings of these preliminary studies suggest that NIR-PIT, when administered as local therapy, may elicit a systemic tumor immune response in the host and sustain the effects of ICIs over an extended period, even in the context of actual clinical practice. Indeed, ongoing clinical trials are evaluating the combination of NIR-PIT and ICI, which is anticipated to make a substantial contribution to the future development of head and neck cancer treatments ^(21)^. Consequently, NIR-PIT is anticipated to elicit a multifaceted antitumor response, encompassing not only physical cell destruction but the induction of tumor immunity. Thus, NIR-PIT represents a promising therapeutic modality for the management of head and neck cancer. However, when considering the therapeutic principle, the efficiency of physical cell destruction with the release of tumor antigens remains an important factor for the induced tumor immune response.

EGFR is frequently expressed in HNSCC ^(22)^, and it is known to promote tumor progression in HNSCC cells through the activation of intracellular signaling pathways. Therefore, EGFR is considered an important therapeutic target in head and neck cancer treatment ^(23)^. In clinical practice, cetuximab, a therapeutic antibody that specifically targets EGFR, has been used for HNSCC in combination with radiation or conventional cytotoxic agents, and its efficacy has been confirmed ^(24)^. While the correlation between EGFR expression and the efficacy of cetuximab therapy remains controversial ^(25)^, several reports have indicated such a correlation ^(26), (27)^, suggesting that EGFR expression in HNSCC is indeed considered an important factor for predicting the treatment effect of cetuximab.

Consequently, cetuximab was selected as the preferred treatment option for head and neck NIR-PIT. For non-SCC to be considered for the treatment procedure, EGFR expression must be confirmed before treatment. In head and neck NIR-PIT, cetuximab, which is light-sensitive, binds to EGFR expressed on the target tumor cells to exert its therapeutic effect. Consequently, the degree of EGFR expression in tumors is directly proportional to the extent of the tumor-destroying effect of NIR-PIT. In the absence of this local destruction of tumor cells, the subsequent tumor immune response should not be triggered. Therefore, a reexamination of the relationship between EGFR as a target antigen and the therapeutic effect of head and neck NIR-PIT is warranted.

A previous clinical study on SCC of the head and neck reported that EGFR expression in tumors was not associated with NIR-PIT ^(16), (28)^; however, the study was conducted with a limited number of patients and did not provide details on how EGFR expression was assessed. Moreover, the paucity of clinical reports examining the relationship between EGFR expression in tumors and the therapeutic effect of NIR-PIT underscores the necessity for further studies to determine whether there is a relationship between EGFR expression and the therapeutic effect of NIR-PIT, including the development of methods for assessing EGFR expression.

In the present study, the intensity of EGFR expression in tumor tissues was found to be associated with the optimal target tumor response of NIR-PIT. This outcome is a natural result of the mechanism of action of NIR-PIT. Conversely, the therapeutic efficacy of NIR-PIT has been attributed to the induction of tumor immunity through the process of ICDs. Consequently, we examined factors reflecting tumor immunity relative to the therapeutic effect of NIR-PIT. The CD4/CD8 ratio, which is indicative of the balance between CD4+ helper T cells and CD8+ cytotoxic T cells within the tumor, serves as a representative factor in this regard. CD4+ helper T cells and CD8+ cytotoxic T cells play important roles in tumor immunity within the tumor microenvironment ^(29), (30)^. The CD4/CD8 ratio has been identified as an important biomarker in cancer therapy, particularly in the context of immunotherapy and chemotherapy, because of its capacity to reflect the equilibrium between these two cell types ^(31)^.

Furthermore, CD4+ T cells are highly diverse, and understanding their polarization is extremely important. Among these, Treg have potent immunosuppressive effects, making it necessary to evaluate the presence of Tregs in the tumor microenvironment ^(32), (33)^. In particular, tumor-infiltrating Tregs are considered a crucial element characterizing the immunosuppressive tumor microenvironment, and an increased frequency of these cells has been reported to correlate with lymph node metastasis and stage in patients with early breast cancer ^(34)^. From these findings, Tregs are considered to be prognostic biomarkers and therapeutic targets in cancer immunotherapy ^(35), (36)^.

In the present study, we examined the therapeutic efficacy of blood biomarkers and NIR-PIT. Blood biomarkers, which are readily obtainable during routine clinical practice, have emerged as a valuable tool in predicting the effectiveness of various cancer treatments because of their inherent advantages, including objectivity and reproducibility ^(37)^. Among the blood biomarkers examined in this study, serum albumin reflects nutritional status and is considered an indicator of disease severity and progression ^(38)^. Serum albumin has also been shown to influence the pharmacodynamics of pharmaceutical agents, such as tyrosine kinase inhibitors, by acting as a transporter for these drugs ^(39), (40)^. Furthermore, serum albumin levels have been used as a predictor of drug efficacy, including ICI, because of its impact on the immune response of the host ^(8), (41)^.

The NLR is an important biomarker in cancer treatment, reflecting the balance between neutrophils and lymphocytes during the immune response. A high NLR indicates a proinflammatory state that may promote tumor growth and metastasis, whereas a low NLR suggests a more favorable immune environment. This ratio has emerged as a prognostic indicator across various cancer types and influences treatment decisions and predicts patient outcomes ^(42)^.

NER reflects the balance between tumor-promoting neutrophils and antitumor eosinophils. Neutrophils facilitate tumor growth and invasion, whereas eosinophils exert their antitumor effects either directly or indirectly. Consequently, a lower NER suggests a more favorable balance for eosinophils, which may enhance the effectiveness of cancer therapy ^(43)^.

Unfortunately, none of these CD4/CD8 ratios, Treg frequency, or blood biomarkers that may reflect tumor immunity showed a clear correlation with the NIR-PIT response in the present study. Nevertheless, these factors may influence the long-term prognosis of NIR-PIT. This is attributable to the fact that, in general, the kinetics of immune-mediated therapy differ from those of conventional therapies, which often exhibit delayed effects without immediate changes in disease progression endpoints ^(44), (45)^. Another finding of the present study is the absence of a correlation between EGFR expression and PFS. This suggests that although EGFR expression contributes to immediate cell destruction by NIR-PIT, additional factors that may involve the tumor immune response may be involved in subsequent progression.

Additionally, in the present study, no clear characteristics were observed between the response and non-response groups regarding patient background factors such as age and tumor volume. Furthermore, while all cases in this study had a PS of 0, preventing a comparison, PS is considered a representative patient factor related to prognosis in ICI treatment ^(6)^. Moving forward, it will be necessary to examine in detail the differences in NIR-PIT therapeutic efficacy based on patient background, not just tissue and blood biomarkers.

The present study had some limitations that should be considered while interpreting the results. First, this was a single-center, retrospective study with a small sample size and short follow-up period.

Specifically, only 13 NIR-PIT cycles on 10 cases were examined in this study, leading to weak statistical power. Therefore, it is not possible to conclude from these results that factors other than EGFR expression are not involved in the efficacy of NIR-PIT. However, considering that EGFR expression showed a strong correlation with the therapeutic efficacy of NIR-PIT despite such a small sample size, it is considered desirable to actively report this result. It is also important to note that blood biomarkers typically correlate with long-term prognosis in immunotherapy. Consequently, the limited follow-up duration in the present study prevents us from establishing a definitive association between NIR-PIT and blood biomarkers.

Second, tumor-related factors, including programmed cell death 1- ligand 1 (PD-L1) expression in tumor tissue, have only been measured in a subset of patients. Consequently, despite the established role of PD-L1 expression in tumors as a significant predictor of immune escape, our study was not designed to assess its influence on clinical outcomes. Third, the present study was designed to examine the relationship between short-term tumor shrinkage effects for each treatment using a sample collected immediately before each treatment cycle; therefore, the examination of OS on a case-by-case basis, including treatment following NIR-PIT, was not possible. Consequently, further studies on biomarkers in patients treated with NIR-PIT are necessary to optimize its efficacy.

## Article Information

### Author Contributions

Shinsuke Suzuki: Conceptualization, design, and writing-original draft preparation. Shinsuke Suzuki, Yukie Taguchi, Haruka Kaya, Takuro Kitabayashi, Yui Miyabe, Nobuko Sato, and Yohei Kawasaki: Data acquisition and interpretation. Hiroshi Nanjo: Pathological examination. Takechiyo Yamada: Supervision. All authors have read and approved the final manuscript.

### Conflicts of Interest

None

### IRB Approval Code and Name of the Institution

Institutional Review Board of Akita University Hospital (No. 3154).

## References

[1] Suzuki M, Kobayashi H, Hanaoka H. Near-infrared photoimmunotherapy for osteosarcoma targeting epidermal growth factor receptor. Transl Oncol. 2024;50:102132. 39357464 10.1016/j.tranon.2024.102132 PMC11471228

[2] Makino T, Sato Y, Uraguchi K, et al. Near-infrared photoimmunotherapy for salivary duct carcinoma. Auris Nasus Larynx. 2024;51(2):323-7. 37775468 10.1016/j.anl.2023.09.006

[3] Hirakawa H, Ikegami T, Kinjyo H, et al. Feasibility of near-infrared photoimmunotherapy combined with immune checkpoint inhibitor therapy in unresectable head and neck cancer. Anticancer Res. 2024;44(9):3907-12. 39197908 10.21873/anticanres.17218

[4] Nishikawa D, Suzuki H, Beppu S, et al. Near-infrared photoimmunotherapy for oropharyngeal cancer. Cancers (Basel). 2022;14(22):5662. 36428754 10.3390/cancers14225662 PMC9688155

[5] Martins F, Sofiya L, Sykiotis GP, et al. Adverse effects of immune-checkpoint inhibitors: epidemiology, management, and surveillance. Nat Rev Clin Oncol. 2019;16(9):563-80. 31092901 10.1038/s41571-019-0218-0

[6] Nishikawa D, Suzuki H, Koide Y, et al. Prognostic markers in head and neck cancer patients treated with nivolumab. Cancers (Basel). 2018;10(12):466. 30477171 10.3390/cancers10120466 PMC6316608

[7] Suzuki S, Abe T, Endo T, et al. Association of pretreatment neutrophil-to-eosinophil ratio with clinical outcomes in patients with recurrent or metastatic head and neck squamous cell carcinoma treated with nivolumab. Cancer Manag Res. 2022;14:3293-302. 36452436 10.2147/CMAR.S382771 PMC9704394

[8] Suzuki S, Taguchi Y, Kitabayashi T, et al. Serum albumin as an independent predictor of long-term survival in patients with recurrent and metastatic head and neck squamous cell carcinoma treated with nivolumab. J Clin Med. 2024;13(9):2456. 38730986 10.3390/jcm13092456 PMC11084251

[9] Meliante PG, Zoccali F, de Vincentiis M, et al. Diagnostic predictors of immunotherapy response in head and neck squamous cell carcinoma. Diagnostics (Basel, Switzerland). 2023;13(5):862. 36900006 10.3390/diagnostics13050862 PMC10001329

[10] Nakajima K, Ogawa M. Near-infrared photoimmunotherapy and anti-cancer immunity. Int Immunol. 2024;36(2):57-64. 37843836 10.1093/intimm/dxad042

[11] Mohiuddin TM, Zhang C, Sheng W, et al. Near infrared photoimmunotherapy: a review of recent progress and their target molecules for cancer therapy. Int J Mol Sci. 2023;24(3):2655. 36768976 10.3390/ijms24032655 PMC9916513

[12] Ishihara H, Nishikawa D, Muraoka D, et al. Changes in serum DAMPs and cytokines/chemokines during near-infrared photoimmunotherapy for patients with head and neck cancer. Cancer Med. 2024;13(1):e6863. 38131639 10.1002/cam4.6863 PMC10807567

[13] Suzuki S, Honda K, Nanjo H, et al. CD147 expression correlates with lymph node metastasis in T1-T2 squamous cell carcinoma of the tongue. Oncol Lett. 2017;14(4):4670-6. 29085466 10.3892/ol.2017.6808 PMC5649530

[14] Mitsunaga M, Ogawa M, Kosaka N, et al. Cancer cell-selective in vivo near infrared photoimmunotherapy targeting specific membrane molecules. Nat Med. 2011;17(12):1685-91. 22057348 10.1038/nm.2554 PMC3233641

[15] Ogawa M, Tomita Y, Nakamura Y, et al. Immunogenic cancer cell death selectively induced by near infrared photoimmunotherapy initiates host tumor immunity. Oncotarget. 2017;8(6):10425-36. 28060726 10.18632/oncotarget.14425 PMC5354669

[16] Bui JD, Suslov N, Yadav D, et al. Intratumoral and peripheral exploratory biomarker analysis in patients with locoregional, recurrent head and neck squamous cell carcinoma (rHNSCC) treated with RM-1929 photoimmunotherapy. Ann Oncol. 2019;30:v470.

[17] Zhang D, Zhao J, Zhang Y, et al. Revisiting immune checkpoint inhibitors: new strategies to enhance efficacy and reduce toxicity. Front Immunol. 2024;15:1490129. 39720720 10.3389/fimmu.2024.1490129 PMC11666542

[18] Khononov I, Jacob E, Fremder E, et al. Hosts. Host response to immune checkpoint inhibitors contributes to tumor aggressiveness. J Immunother Cancer. 2021;9(3):e001996. 33707313 10.1136/jitc-2020-001996 PMC7957134

[19] Gulati S, Crist M, Riaz MK, et al. Durvalumab plus cetuximab in patients with recurrent or metastatic head and neck squamous cell carcinoma: an open-label, nonrandomized, Phase II clinical trial. Clin Cancer Res. 2023;29(10):1906-15. 36802410 10.1158/1078-0432.CCR-22-3886 PMC10192200

[20] Nagaya T, Friedman J, Maruoka Y, et al. Hosts. Host immunity following near-infrared photoimmunotherapy is enhanced with PD-1 checkpoint blockade to eradicate established antigenic tumors. Cancer Immunol Res. 2019;7(3):401-13. 30683733 10.1158/2326-6066.CIR-18-0546 PMC8237708

[21] Zou J, Li L, Yang Z, et al. Phototherapy meets immunotherapy: a win-win strategy to fight against cancer. Nanophotonics. 2021;10(12):3229-45.

[22] Kalyankrishna S, Grandis JR. Epidermal growth factor receptor biology in head and neck cancer. J Clin Oncol. 2006;24(17):2666-72. 16763281 10.1200/JCO.2005.04.8306

[23] Johnson DE, Burtness B, Leemans CR, et al. Head and neck squamous cell carcinoma. Nat Rev Dis Primers. 2020;6(1):92. 33243986 10.1038/s41572-020-00224-3 PMC7944998

[24] Bonner JA, De Los Santos J, Waksal HW, et al. Epidermal growth factor receptor as a therapeutic target in head and neck cancer. Semin Radiat Oncol. 2002;12(3)(suppl 2):11-20. 12174340 10.1053/srao.2002.34864

[25] Labianca R, La Verde N, Garassino MC. Development, and clinical indications of cetuximab. Int J Biol Markers. 2007;22(4)(1 suppl 4):40-6. 28207113 10.1177/17246008070221s405

[26] Smilek P, Dusek L, Veselý K, et al. Correlation of expression of Ki-67, EGFR, c-erbB-2, MMP-9, p53, bcl-2, CD34 and cell cycle analysis with survival in head and neck squamous cell cancer. J Exp Clin Cancer Res. 2006;25(4):549-55. 17310847

[27] Hartmann S, Seher A, Brands RC, et al. Influence of epidermal growth factor receptor expression on the cetuximab and panitumumab response rates of head and neck carcinoma cells. J Craniomaxillofac Surg. 2014;42(7):1322-8. 24780353 10.1016/j.jcms.2014.03.018

[28] Cognetti DM, Johnson JM, Curry JM, et al. Phase 1/2a, open-label, multicenter study of RM-1929 photoimmunotherapy in patients with locoregional, recurrent head and neck squamous cell carcinoma. Head Neck. 2021;43(12); (March):3875-87. 34626024 10.1002/hed.26885 PMC9293150

[29] Speiser DE, Chijioke O, Schaeuble K, et al. CD4+ T cells in cancer. Nat Cancer. 2023;4(3):317-29. 36894637 10.1038/s43018-023-00521-2

[30] Farhood B, Najafi M, Mortezaee K. CD8+ cytotoxic T lymphocytes in cancer immunotherapy: a review. J Cell Physiol. 2019;234(6):8509-21. 30520029 10.1002/jcp.27782

[31] Albarrán Fernández V, Ballestín Martínez P, Stoltenborg Granhøj J, et al. Biomarkers for response to TIL therapy: a comprehensive review. J Immunother Cancer. 2024;12(3):e008640. 38485186 10.1136/jitc-2023-008640 PMC10941183

[32] Afzali B, Lombardi G, Lechler RI, et al. The role of T helper 17 (Th17) and regulatory T cells (Treg) in human organ transplantation and autoimmune disease. Clin Exp Immunol. 2007;148(1):32-46. 17328715 10.1111/j.1365-2249.2007.03356.x PMC1868863

[33] Curiel TJ. Tregs and rethinking cancer immunotherapy. J Clin Invest. 2007;117(5):1167-74. 17476346 10.1172/JCI31202 PMC1857250

[34] Solis-Castillo LA, Garcia-Romo GS, Diaz-Rodriguez A, et al. Tumor-infiltrating regulatory T cells, CD8/Treg ratio, and cancer stem cells are correlated with lymph node metastasis in patients with early breast cancer. Breast Cancer. 2020;27(5):837-49. 32180141 10.1007/s12282-020-01079-y

[35] Saleh R, Elkord E. FoxP3+ T regulatory cells in cancer: prognostic biomarkers and therapeutic targets. Cancer Lett. 2020;490:174-85. 32721551 10.1016/j.canlet.2020.07.022

[36] Tay C, Tanaka A, Sakaguchi S. Tumor-infiltrating regulatory T cells as targets of cancer immunotherapy. Cancer Cell. 2023;41(3):450-65. 36917950 10.1016/j.ccell.2023.02.014

[37] Xu-Welliver M, Carbone DP. Blood-based biomarkers in lung cancer: prognosis and treatment decisions. Transl Lung Cancer Res. 2017;6(6):708-12. 29218272 10.21037/tlcr.2017.09.08 PMC5709136

[38] Yeun JY, Kaysen GA. Factors influencing serum albumin in dialysis patients. Am J Kidney Dis. 1998;32(6)(suppl 4):S118-25. 10.1016/s0272-6386(98)70174-x 9892378

[39] Ikeda S, Yoshioka H, Ikeo S, et al. Serum albumin level as a potential marker for deciding chemotherapy or best supportive care in elderly, advanced non-small cell lung cancer patients with poor performance status. BMC Cancer. 2017;17(1):797. 29183294 10.1186/s12885-017-3814-3 PMC5704395

[40] Cai W, Kong W, Dong B, et al. Pretreatment serum prealbumin as an independent prognostic indicator in patients with metastatic renal cell carcinoma using tyrosine kinase inhibitors as first-line target therapy. Clin Genitourin Cancer. 2017;15(3):e437-46. 28188047 10.1016/j.clgc.2017.01.008

[41] Zheng M. Serum albumin: a pharmacokinetic marker for optimizing treatment outcome of immune checkpoint blockade. J Immunother Cancer. 2022;10(12):e005670. 36600664 10.1136/jitc-2022-005670 PMC9772729

[42] Cupp MA, Cariolou M, Tzoulaki I, et al. Neutrophil to lymphocyte ratio and cancer prognosis: an umbrella review of systematic reviews and meta-analyses of observational studies. BMC Med. 2020;18(1):360. 33213430 10.1186/s12916-020-01817-1 PMC7678319

[43] Sahin TK, Ayasun R, Rizzo A, et al. Prognostic value of neutrophil-to-eosinophil ratio (NER) in cancer: a systematic review and meta-analysis. Cancers (Basel). 2024;16(21):3689. 39518127 10.3390/cancers16213689 PMC11545344

[44] Gulley JL, Madan RA, Pachynski R, et al. Role of antigen spread and distinctive characteristics of immunotherapy in cancer treatment. J Natl Cancer Inst. 2017;109(4):djw261. 28376158 10.1093/jnci/djw261 PMC5441294

[45] Hoos A. Evolution of end points for cancer immunotherapy trials. Ann Oncol. 2012;23(suppl 8):viii47-52. 22918928 10.1093/annonc/mds263

